# Dual ankyrinG and subpial autoantibodies in a man with well-controlled HIV infection with steroid-responsive meningoencephalitis: A case report

**DOI:** 10.3389/fneur.2022.1102484

**Published:** 2023-01-23

**Authors:** Christopher M. Bartley, Thomas T. Ngo, Cathryn R. Cadwell, Adil Harroud, Ryan D. Schubert, Bonny D. Alvarenga, Isobel A. Hawes, Kelsey C. Zorn, Trung Hunyh, Lindsay H. Teliska, Andrew F. Kung, Shailee Shah, Jeffrey M. Gelfand, Felicia C. Chow, Matthew N. Rasband, Divyanshu Dubey, Sean J. Pittock, Joseph L. DeRisi, Michael R. Wilson, Samuel J. Pleasure

**Affiliations:** ^1^Weill Institute for Neurosciences, University of California, San Francisco, San Francisco, CA, United States; ^2^Department of Psychiatry and Behavioral Sciences, University of California, San Francisco, San Francisco, CA, United States; ^3^Department of Neurological Surgery, University of California, San Francisco, San Francisco, CA, United States; ^4^Department of Pathology, University of California, San Francisco, San Francisco, CA, United States; ^5^Department of Neurology, University of California, San Francisco, San Francisco, CA, United States; ^6^Biomedical Sciences Graduate Program, University of California, San Francisco, San Francisco, CA, United States; ^7^Department of Biochemistry and Biophysics, University of California, San Francisco, San Francisco, CA, United States; ^8^Department of Neuroscience, Baylor College of Medicine, Houston, TX, United States; ^9^School of Medicine, University of California, San Francisco, San Francisco, CA, United States; ^10^Department of Neurology, Vanderbilt University Medical Center, Nashville, TN, United States; ^11^Center for Multiple Sclerosis and Autoimmune Neurology, Mayo Clinic, Rochester, MN, United States; ^12^Department of Neurology, Mayo Clinic Foundation, Rochester, MN, United States; ^13^Department of Laboratory Medicine and Pathology, Mayo Clinic Foundation, Rochester, MN, United States; ^14^Chan Zuckerberg Biohub, San Francisco, CA, United States

**Keywords:** meningoencephalitis, axon initial segment (AIS), node of Ranvier, human immunodeficiency virus (HIV), ankyrinG, ANK3, autoantibody, autoimmune

## Abstract

Neuroinvasive infection is the most common cause of meningoencephalitis in people living with human immunodeficiency virus (HIV), but autoimmune etiologies have been reported. We present the case of a 51-year-old man living with HIV infection with steroid-responsive meningoencephalitis whose comprehensive pathogen testing was non-diagnostic. Subsequent tissue-based immunofluorescence with acute-phase cerebrospinal fluid revealed anti-neural antibodies localizing to the axon initial segment (AIS), the node of Ranvier (NoR), and the subpial space. Phage display immunoprecipitation sequencing identified ankyrinG (AnkG) as the leading candidate autoantigen. A synthetic blocking peptide encoding the PhIP-Seq-identified AnkG epitope neutralized CSF IgG binding to the AIS and NoR, thereby confirming a monoepitopic AnkG antibody response. However, subpial immunostaining persisted, indicating the presence of additional autoantibodies. Review of archival tissue-based staining identified candidate AnkG autoantibodies in a 60-year-old woman with metastatic ovarian cancer and seizures that were subsequently validated by cell-based assay. AnkG antibodies were not detected by tissue-based assay and/or PhIP-Seq in control CSF (*N* = 39), HIV CSF (*N* = 79), or other suspected and confirmed neuroinflammatory CSF cases (*N* = 1,236). Therefore, AnkG autoantibodies in CSF are rare but extend the catalog of AIS and NoR autoantibodies associated with neurological autoimmunity.

## Introduction

Immunocompromised individuals are vulnerable to neuroinvasive pathogens that can cause meningitis, encephalitis, and myelitis. Infections of the central nervous system (CNS) are a leading cause of death in persons with acquired immunodeficiency syndrome (AIDS) but are uncommon in those with well-controlled HIV. Yet, because CNS infection may still occur in well-controlled HIV (1, 2), comprehensive microbial testing and empiric antibiotics are warranted in patients with signs and symptoms consistent with CNS infection. However, ongoing neuroinflammation, despite comprehensive and persistently negative pathogen testing, presents a clinical dilemma.

Other causes of neuroinflammation in persons with well-controlled HIV include macrophage-mediated HIV encephalitis and CD8 encephalitis (3)—a hallmark of both of which is CNS HIV escape, in which HIV RNA can be detected in the CSF (4). Persons living with HIV are also at an increased risk for immune dysregulation (5, 6) and autoimmune disorders of the nervous system including acute and chronic inflammatory demyelinating polyneuropathy (7) (AIDP and CIDP, respectively).

Here, we present the case of a man with well-controlled HIV who developed steroid-responsive meningoencephalitis in the absence of HIV escape. Subsequent research studies identified a novel anti-ankyrinG (AnkG) antibody and a second antibody directed at a subpial antigen in his CSF. A search of archival neuroinflammatory cases identified an HIV-negative woman with a history of cancer and seizures who harbored the same anti-neuronal antibody but not the subpial antibody, indicating that AnkG may in rare cases be the target autoantigen in seronegative autoimmune neurological syndromes.

## Case presentations

### Case 1

**Figure 1:**
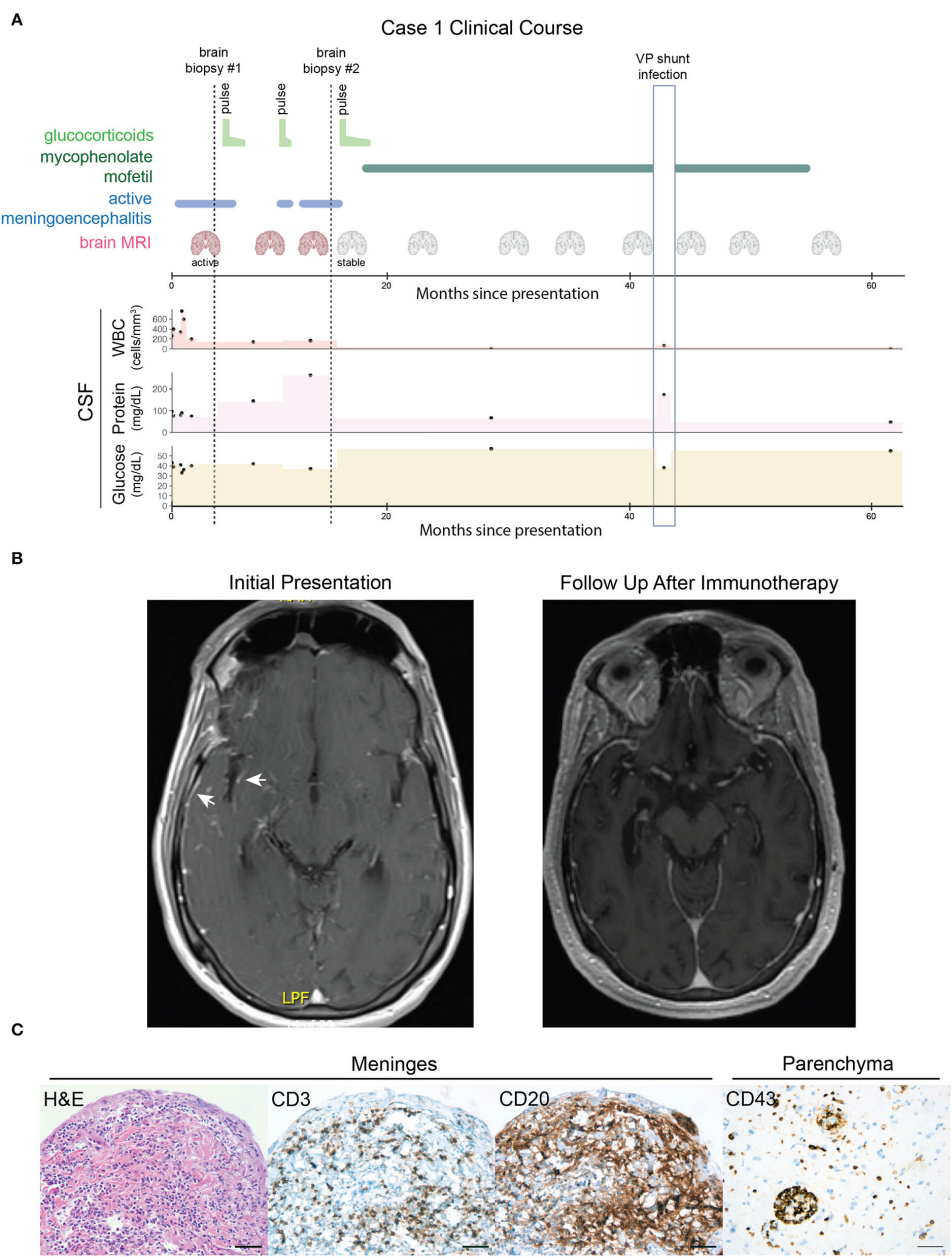
Case 1, course of illness and clinical studies. **(A)** A graphical time course of events. Brain biopsies are indicated by vertical dotted lines. Periods of glucocorticoid and mycophenolate mofetil immunosuppressive therapy are indicated by light and dark green lines, respectively. Active meningoencephalitis is indicated by the blue line. MRI scans are indicated by the coronal brain icon (red = active meningoencephalitis and gray = stable/no enhancement or worsening of the encephalitic process). Black dots indicate clinical CSF values for white blood count (WBC, ref. ≤5 cells/μL), protein (ref. ≤60 mg/dL), and glucose levels (ref. ≥50 and ≤80 mg/dL). Colored areas between dots have been added to aid visual assessment. The vertical light blue outlined box indicates a period of acute illness secondary to a confirmed ventriculoperitoneal (VP) shunt infection resulting in a 2-month interruption in mycophenolate mofetil treatment and transient CSF abnormalities thought to be unrelated to the autoimmune meningoencephalitis. **(B)** Axial T1 post-gadolinium MRIs demonstrate that the right temporal lobe and insular leptomeningeal enhancement present at initial presentation (arrows, left image) had resolved at a follow-up appointment after immunosuppressive therapy (right image). **(C)** Histopathology findings: hematoxylin and eosin (H&E) staining of a biopsy of the meninges revealed a dense, mixed inflammatory infiltrate. Immunohistochemistry of the meninges identified scattered CD3+ T cells and scattered CD20+ B cells. A separate biopsy of the brain parenchyma revealed perivascular and intraparenchymal collections of CD43+ inflammatory cells. Scale bars = 50 μm.

A 51-year-old man with well-controlled HIV (CD4^+^ count 791 cells/μL and undetectable viral load) on tenofovir alafenamide, emtricitabine, and dolutegravir presented to the emergency room with several weeks of headache, right-sided hearing loss, and memory impairment. He smoked one pack of cigarette per day and marijuana but did not use other drugs. He had lived in the San Francisco Bay Area for over 20 years and was an avid gardener. He did not have a history of incarceration, homelessness, or international travel (Figure 1A).

In the emergency department, he had a witnessed seizure and an MRI of the brain showed swelling of the right cerebral cortex, with abnormal diffusion, T2/FLAIR hyperintensity, and patchy enhancement, and without abnormal susceptibility weighted imaging (Figure 1B). The imaging was consistent with meningoencephalitis, and serial lumbar punctures (LP) revealed a neutrophil-predominant pleocytosis (WBC: 260–756/μL with 12–70% neutrophils), mild hypoglycorrhachia (nadir 33 mg/dL), and elevated protein (76–95 mg/dL). Gram stain and bacterial culture in CSF were negative, as was cryptococcal antigen. He was started on empiric valacyclovir and broad-spectrum antibiotics.

After 3 weeks of inpatient antimicrobial treatment, the patient failed to improve. Comprehensive serological and cerebrospinal fluid (CSF) testing for infectious etiologies was non-diagnostic including serum toxoplasmosis IgG and CSF fungal cultures, microbial antigen and antibody testing, VDRL, universal bacterial, fungal, and mycobacterial PCR, and metagenomic next-generation sequencing (mNGS). Diagnostic biopsy of the leptomeninges overlying the right cerebrum revealed dense connective tissue with a mixed inflammatory infiltrate including granulocytes, and B and T cells (Figure 1C), suggestive of an infectious etiology. The underlying cortical brain tissue was also biopsied and showed no significant pathological abnormality. Histochemical stains for acid-fast bacillus (AFB), Grocott's methenamine silver stain (GMS), and period acid-Schiff (PAS) stains on the brain tissue were negative, arguing against mycobacterial or fungal infection and Whipple's disease. A Quantiferon Gold TB test was negative. However, given the insensitivity of CNS TB diagnostics, the lack of clinical improvement, and a persistent neutrophil-predominant CSF pleocytosis with mild hypoglycorrhachia, he was started on empiric therapy for TB meningoencephalitis with isoniazid, rifampin, ethambutol, and pyrazinamide and transferred to an outpatient rehabilitation facility.

One week after discharge (week 7), the patient presented to the emergency department with new-onset expressive aphasia, hyponatremia, and ataxia. EEG revealed right greater than left diffuse background slowing and disorganization without epileptiform activity. Repeat LP again showed a neutrophilic pleocytosis (WBC 194/μL with 50% neutrophils), protein 75 mg/dL, elevated IgG index (0.7, ref. ≤0.6), and five restricted oligoclonal bands (OCBs). Brain MRI showed persistent meningoencephalitis with new ventriculomegaly. A large volume LP was performed. The opening pressure was 25 cm and the patient regained his speech after 1 h later, had improved gait, and improved mental status. He proceeded to get a ventriculoperitoneal shunt and continued on anti-TB therapy.

A CSF autoimmune encephalopathy panel was reported as positive for NMDA receptor autoantibodies (1:8 tissue titer) as well as putatively non-specific additional staining on tissue. The whole body PET and scrotal ultrasound were negative. A repeat autoimmune encephalopathy panel was NMDA receptor antibody positive on cell-based assay but negative on reflex tissue staining. Because the clinical phenotype was markedly inconsistent with anti-NMDAR encephalitis, empiric anti-TB therapy was initially continued. Nonetheless, an autoimmune etiology was increasingly considered as a result of the repeatedly negative infectious workup and lack of improvement in antibiotic therapy. In this context, he received a 5-day course of methylprednisolone 1 gram daily on week 8 followed by a prednisone taper with dramatic improvement in his memory and language. Anti-TB therapy was ultimately discontinued after ~1 month of treatment.

Over the following months, attempts to taper off prednisone resulted in clinical and radiological deterioration. Surveillance brain MRIs demonstrated worsening of meningoencephalitis with a new focus on enhancement in the left amygdala. A second brain biopsy of glucocorticoids at month 10 showed severe granulomatous and suppurative meningoencephalitis with a perivascular and intraparenchymal lymphocytic infiltrate, diffuse astrogliosis, and microglial activation. The pattern of inflammation was again suspicious for mycobacterial infection; however, AFB and AFB-FITE stains and AFB culture of the brain tissue were negative, as were mycobacterial universal PCR and metagenomic next-generation sequencing.

Three weeks after the second biopsy, the patient was retreated with IV methylprednisolone followed by oral prednisone maintenance with subsequent clinical improvement over the next few months. Repeat imaging 3 months after the resumption of glucocorticoid treatment demonstrated near the total resolution of leptomeningeal enhancement and non-progression of his encephalitis. The patient was then transitioned empirically to mycophenolate mofetil and successfully weaned off prednisone without recurrence of his meningoencephalitis. He remained seizure-free on antiseizure therapy. Repeat CSF at 26 months post-presentation showed normal cellularity (3 WBC/μL with 87% lymphocytes and 17% monocytes), normal glucose (57 mg/dL), mildly elevated protein (67 mg/dL), persistent elevated IgG index (0.7 ref. ≤0.6), and two oligoclonal bands. MRI brain every 6 months showed stable multifocal T2/FLAIR cortical and white matter hyperintensities without associated enhancement. Following 3 years of immunosuppressive therapy, 4.5 years after his initial presentation, mycophenolate mofetil was stopped and the patient remained stable. Neurological assessment documented fixed sequelae consisting of dysarthria, executive dysfunction, and episodic memory impairment.

### Case 2

A 60-year-old woman with a history significant for smoking, non-insulin-dependent type II diabetes, renal cell carcinoma status post-left radical nephrectomy and node dissection and essential hypertension, and no family history of autoimmune neurologic or oncologic disease was diagnosed with stage IIIC metastatic ovarian cancer. She underwent bilateral salpingoopherectomy and supracervical hysterectomy, and carboplatin and taxol therapy. Two years later, in the context of recurrent ovarian cancer again undergoing carboplatin, taxol, and avastin therapy, she developed seizures with interictal abnormalities noted in the left frontotemporal region on EEG. Her MRI at the time had bilateral parieto-occipital FLAIR abnormalities without evidence of meningeal involvement. Her spinal fluid was notable for elevated protein. Her seizures were attributed to posterior reversible encephalopathy syndrome (PRES) in the setting of carboplatin use. Six years after her initial diagnosis, she had a recurrence of her ovarian cancer. She was placed on tamoxifen, and over the next 18 months, she was treated with topotecan and bevacizumab (15 cycles), paclitaxel (five cycles), gemcitabine (two doses), and doxorubicin. Eight years after her initial diagnosis, she again developed seizures characterized by unresponsiveness, rightward head and eye deviation, and right upper extremity tonic–clonic movements. EEGs at the time captured left temporal seizures and interictal abnormalities and were treated with levetiracetam. An MRI brain with contrast at the time showed non-specific ischemic changes without metastatic disease. Her spinal fluid was non-inflammatory, and autoantibody testing at the time was negative. Her CSF protein was elevated (100 mg/dL), and oligoclonal bands were not sent. Four months later, she was admitted to the hospital with neutropenic fever (absolute neutrophil count 0.3), mouth sores, and dysphagia, and treated for sepsis. Due to ongoing failure to thrive, the patient elected to pursue hospice and she subsequently died.

## Results

### Identification of anti-neuronal autoantibodies by tissue-based assay

**Figure 2:**
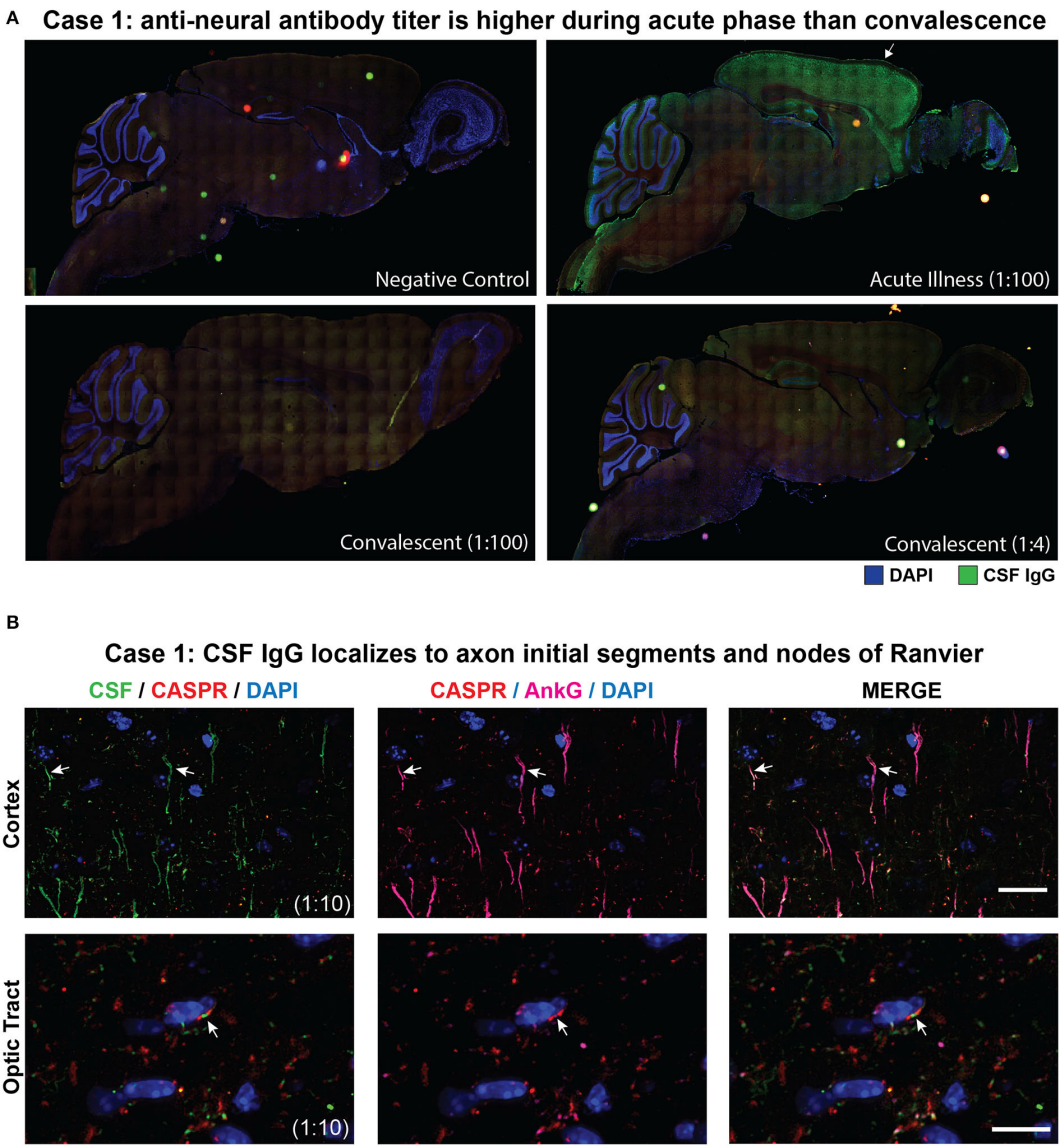
Tissue-based immunofluorescence (TBIF) assays. **(A)** Panoramic images of sagittal mouse brain TBIF with acute-phase CSF at a 1:100 dilution or convalescent CSF at a 1:4 dilution. **(B)** Colocalization of case 1 acute-phase CSF with AnkG at the AIS in the cortex (arrows, top row) and NoR in the optic tract (arrows, bottom row). Nodal CSF IgG localizes to the NoR delimited by paranodal CASPR (red). Scale bars = 10 μm.

CSF from cases 1 and 2 was screened for autoantibodies by tissue-based immunofluorescence (TBIF). At a 1:100 dilution, acute-phase CSF from case 1 produced a pattern of immunoreactivity that localized to axon initial segment (AIS)-like structures throughout the cortex and node of Ranvier (NoR)-like structures in the optic tract. In contrast, case 1's convalescent CSF was not immunoreactive at a 1:100 dilution and stained only weekly at a 1:4 dilution, suggesting a decrease in the autoantibody titer following immunosuppressive therapy (Figure 2A).

To verify immunoreactivity to the AIS and NoR, we immunostained mouse brain tissue with acute-phase CSF and commercial antibodies to the AIS and nodal marker AnkG and the paranodal marker protein contactin-associated protein 1 (CASPR). Case 1 CSF IgG colocalized with AnkG at the AIS and NoR, thereby confirming AIS-NoR immunoreactivity (Figure 2B). A review of TBIF results from a separate study revealed that none of 46 CSF samples from people living with HIV produced AIS/NoR immunostaining (8).

Case 2 was identified as part of a screen for TRIM46 autoimmunity (9). CSF was screened at a 1:4 dilution and produced a similar AIS and NoR-like pattern of immunoreactivity throughout the murine brain (Supplementary Figure 1A) but was negative for TRIM46 on the cell-based assay. Owing to limited sample volume, co-staining with commercial AnkG and CASPR antibodies was not performed, and the remaining CSF was reserved for candidate autoantigen discovery and validation.

We did not identify another case of anti-AIS/NoR immunoreactivity among the 39 control and 1,236 candidate CNS neuroinflammatory patients screened by TBIF in our laboratory.

### PhIP-Seq identifies AnkG as case 1's putative autoantigen

**Figure 3:**
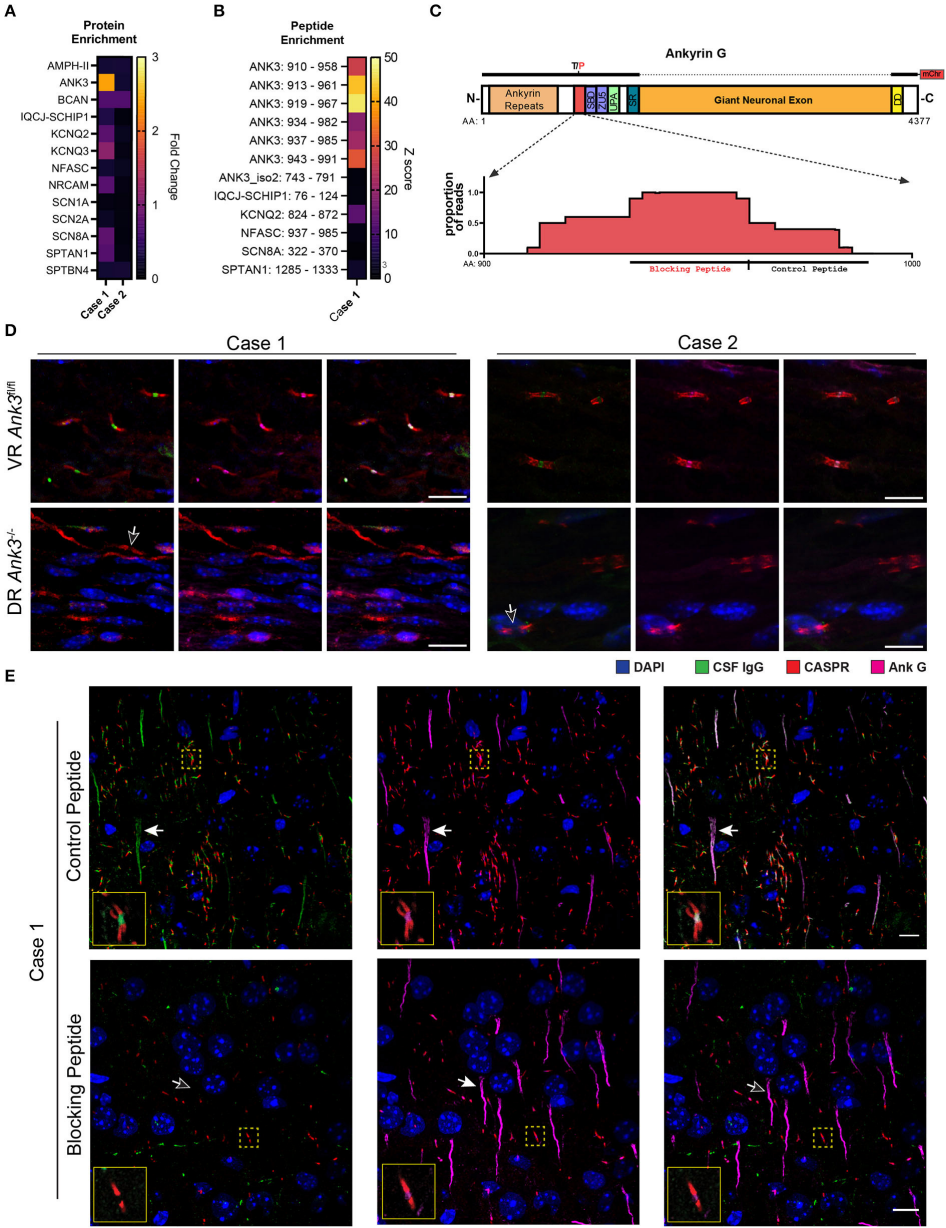
Validation of AnkG autoantibodies in the CSF of cases 1 and 2. **(A)** A heatmap of the fold change of PhIP-Seq AIS and NoR protein enrichments for cases 1 and 2 CSF relative to control PhIP-Seq runs (*N* = 4,216, comprised of beads only and healthy control CSF and serum). **(B)** Heatmap of case 1 acute-phase CSF AIS and NoR peptides enriched with ≥10-fold above controls (as above) in both technical replicates relative to the same controls as above. Individual peptides are annotated as “GENE: amino acid range” (e.g., ANK3: 910–958). Enrichment values are expressed as Z-scores. **(C)** Model of AnkG, its protein domains, and the case 1 target epitope. SBD, spectrin-binding domain (first ZU5 domain); UPA, UPA domain; SR, serine rich; DD, death domain. Above the model is a representation of the 270 kDa rat AnkG-mCherry construct used for the CBAs (T/P indicates that rats encode a proline residue instead of the threonine residue present in humans and mice). The dotted line indicates that the giant neuronal exon is absent in the 270 kDa construct. Below the model is a pileup graph of the position of residues encoded by AnkG-enriched peptides mapped to human AnkG (Uniprot.org Q12955-3). The relative positions of the synthetic blocking and control peptides used in TBIF experiments are indicated below the pileup map. **(D)** Cases 1 and 2 CSF immunostaining of AnkG-expressing ventral roots (VR-*Ank3*^*fl*/*fl*^) and AnkG-deficient dorsal roots (DR-*Ank3*^−/−^) from *Advillin*^*Cre*/+^ and *Ank3*^*flox*/*flox*^ mice. The white outlined arrow indicates the absence of nodal immunostaining by CSF IgG (green). **(E)** Immunostaining of mouse cortex with case 1 CSF after preabsorption with the control peptide (top row) or blocking peptide (lower row). Filled arrows indicate the AIS, whereas the unfilled arrow indicates the absence of CSF IgG immunostaining of the AIS in the blocking peptide condition. The solid yellow outlined insets show the NoRs in the yellow dotted squares at higher magnification. Scale bar = 10 μm.

To identify candidate anti-AIS/NoR autoantibodies, we screened both cases in technical replicate by phage display immunoprecipitation sequence (PhIP-Seq) (10). We restricted our analysis to AIS/NoR proteins (as defined by EMBL-EBI, see Supplementary methods) and normalized total protein enrichments to 4,215 control samples (comprised of beads only and healthy CSF and serum including replicates). Case 1 nominally enriched AnkG 2.4-fold above controls (Z-score of 0.6); however, case 2 failed to enrich any AIS/NoR proteins (Figure 3A).

We hypothesized that spurious enrichment of non-target peptides could attenuate the contribution of individual target peptides to total protein enrichment values. Therefore, we filtered for AIS/NoR peptides that were enriched at least 10-fold in both technical replicates. For case 1, more than half of enriched peptides mapped to ankyrinG, six of which shared an epitope and had Z-scores ranging between 18 and 47 (Figure 3B). Multiple sequence alignment indicated that case 1's putative epitope lays within amino acids (AA) 937–958 (Figure 3C).

Because of case 1's HIV status, we reviewed PhIP-Seq data from 33 HIV CSF samples (*N* = 15 well-controlled and *N* = 18 untreated HIV) and found that neither AnkG nor epitope AnkG^937−958^ were enriched above negative controls (8). Furthermore, a BLAST search revealed that this epitope does not harbor sequence similarity to HIV arguing against molecular mimicry. Although NMDAR antibodies have been reported in anti-GFAP autoimmune meningoencephalitis (11), GFAP peptides were not observed in case 1's PhIP-Seq data despite being enriched by a commercial polyclonal antibody to GFAP as a positive control (data not shown).

In contrast to case 1, a peptide-level analysis of case 2 PhIP-Seq data failed to identify significant AIS/NoR peptide enrichments (data not shown) suggesting that case 2's autoantibodies recognize conformational or post-translationally modified epitopes that are not represented in our PhIP-Seq library.

### Cell-based assay (CBA) identifies AnkG as case 2's putative autoantigen

Surprisingly, case 1 failed to bind rat-mCherry-AnkG (data not shown). However, a comparison of human, rat, and murine ankyrinG revealed that rat AnkG differs from human and murine AnkG by a single amino acid (proline in rat vs. threonine in human and mouse) in the center of the putative AnkG^937−958^ epitope (Figure 3C).

Based on the PhIP-Seq and TBIF findings, we hypothesized that case 2's target epitopes were conformational or post-translationally modified. We screened case 2's CSF against previously established AnkG and βIV spectrin variant *Σ*1 and *Σ*6 HEK 293T cell-based assays (12). We found that case 2 was strongly immunoreactive to overexpressed rat-mCherry-AnkG (Supplementary Figure 1B) but not βIV spectrin *Σ*1 or *Σ*6 (data not shown).

### Validation of AnkG autoantibodies in both cases by conditional knockout tissue-based assay

To confirm anti-AnkG antibodies in both cases, we immunostained conditional AnkG knockout mice with patient's CSF. The *Advillin* promoter is active in dorsal sensory neurons but inactive in ventral motor neurons (13). Therefore, we immunostained ventral root motor neurons (VR-*Ank3*^*flfl*^) and dorsal root sensory neurons (DR-*Ank3*^−/−^) from *Advillin-Cre:Ank3*^*fl*/*fl*^ mice with CSF from cases 1 and 2. In both cases, CSF IgG localized to VR-*Ank3*^*flfl*^ NoR but not DR-*Ank3*^−/−^ NoR, thereby confirming that the staining was due to the presence of AnkG autoantibodies in both cases' CSF samples (Figure 3D).

### Case 1's anti-AnkG antibodies bind exclusively within the PhIP-Seq-predicted epitope

We hypothesized that case 1's anti-AnkG antibody population was monoepitopic because AnkG PhIP-Seq-enriched peptides shared a common epitope. Although PhIP-Seq lacks conformational and post-translational epitopes, the negative cell-based assay using rat AnkG further suggested that case 1's autoantibody binding was restricted to amino acid 937–958.

To test for monoepitopic antibody binding, we commercially synthesized a peptide encoding case 1's putative epitope (blocking peptide: AnkG^934−961^) and a peptide encoding the adjacent C-terminal 25 AA as a non-epitope peptide control (control peptide: AnkG^962−989^) (Figure 3C).

In technical quadruplicate, we immunostained rodent brain tissue with acute-phase CSF without peptide, or after pre-incubation with control or blocking peptide in five molar excess. Preincubation of case 1 CSF with the blocking peptide, but not the control peptide, completely abrogated CSF IgG binding to both the AIS and NoR without affecting the binding of a commercial AnkG antibody (NeuroMab N106/36) that binds outside of the control and blocking epitopes (Figure 3E).

### Case 1 harbors a distinct autoantibody population to a subpial antigen

**Figure 4:**
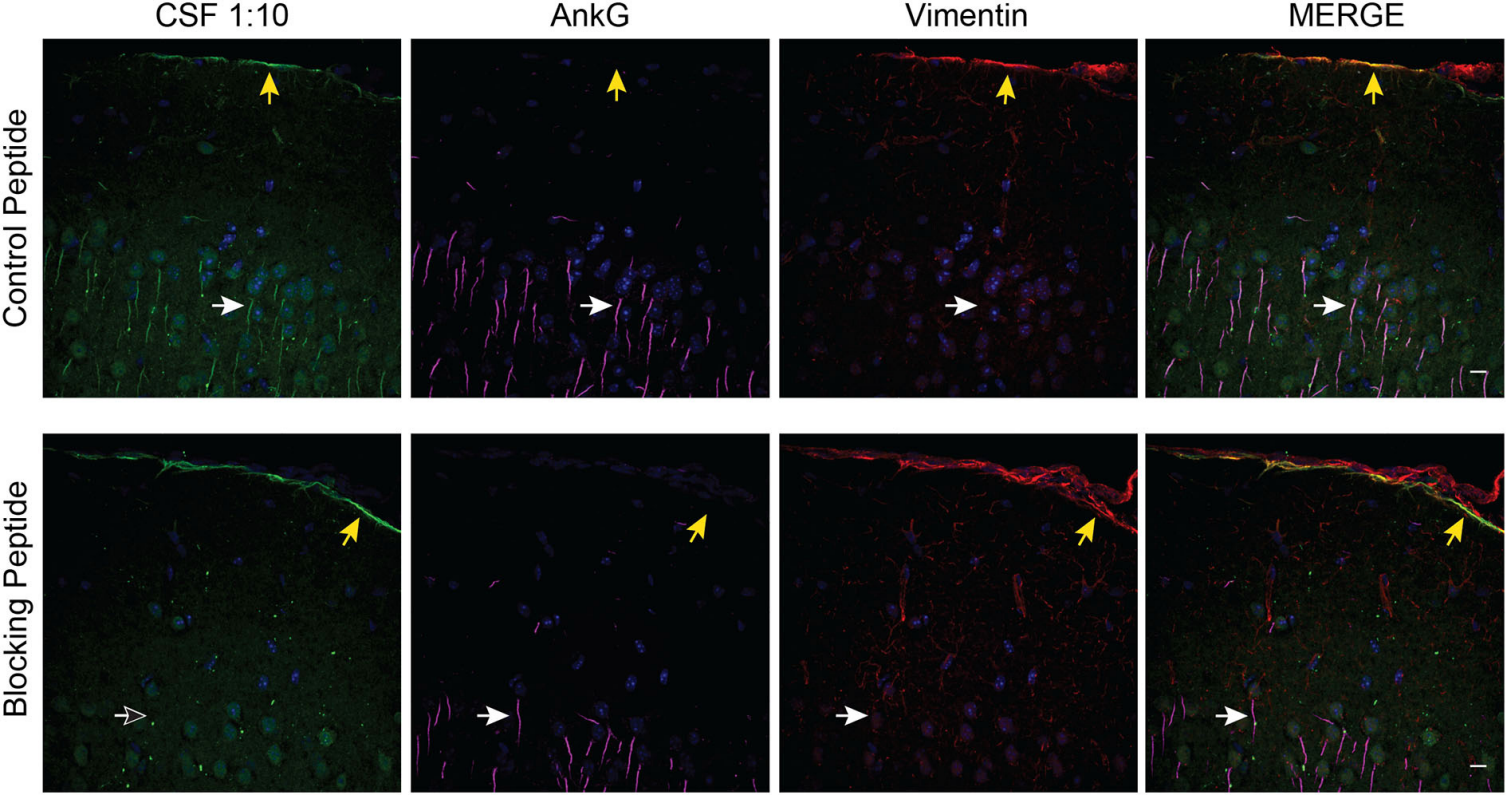
Identification of a subpial autoantibody. Case 1 CSF TBIF of murine cortex with control or blocking peptide. Tissue was co-stained with AnkG (magenta) and Vimentin (red) to identify the overlying pial tissue. Yellow arrows point to subpial CSF IgG. White arrows point to an AIS. The empty arrow in the lower left panel indicates the absence of AIS immunostaining. Scale bar = 10 μm.

We observed that the AnkG blocking peptide abrogated AIS and NoR immunoreactivity but not the limited nuclear and subpial CSF IgG immunoreactivity (Figure 4). However, a review of the PhIP-Seq data failed to yield an obvious candidate antigen that would explain the subpial immunostaining.

## Discussion

This case demonstrates the complexity of diagnosis and clinical management of people living with well-controlled HIV who present with neuroinflammatory syndromes. Although broad-based testing of blood, CSF, and brain tissue for infections was consistently negative, it is possible that a preceding CNS infection was the precipitant of this patient's autoimmune meningoencephalitis. Nonetheless, this case also highlights the need for continued autoantibody discovery in seronegative autoimmune CNS syndromes to decrease the time to treatment.

Despite negative infectious testing, case 1's low CSF glucose sustained concern for an undetected microorganism. Hypoglycorrhachia is common in infectious meningitis and is unlikely to be due to pleocytosis alone in aseptic meningitis except for specific non-infectious syndromes such as systemic lupus erythematosus (14), which case 1 did not have. Hypoglycorrhachia can also be observed in neurosarcoidosis, but the absence of sarcoidosis signs and symptoms on physical examination, laboratory studies, body imaging acutely, and longitudinal yearly followup were inconsistent with this diagnosis. Low CSF glucose has also been reported in autoimmune meningoencephalitis due to checkpoint inhibitor therapy (15–17) and GFAP meningoencephalitis (18)—the most common known autoantigen associated with autoimmune meningoencephalits (11). Although we did not test for GFAP antibodies by CBA, our case did not show GFAP-like staining by TBIF.

Although IgG class NMDAR autoantibodies were detected in the CSF of our patient, they were low titer and inconsistently detected on clinical testing; this, along with the lack of typical clinical features of anti-NMDAR encephalitis, led us to conclude that these represented false-positive anti-NMDAR testing, which is known to occur (19). NMDAR antibodies have also been observed in the setting of HIV escape (20), infectious meningoencephalitis (21, 22), and GFAP astrocytopathy (11). However, in these cases, as in ours, the phenotype was atypical for NMDAR encephalitis.

Instead, research testing identified a high-titer antibody to AnkG that decreased in titer during convalescence. AnkG is enriched in the AIS and NoR, subcellular structures with similar molecular profiles that are targeted by autoantibodies in diverse autoimmune neurological disorders including antibodies that bind to gangliosides (23), neurofascins (24, 25), CASPR (26), CASPR2 (27, 28), TRIM46 (9, 29), and βIV-spectrin (12). Grouping autoantibody-mediated neurological patients by target epitope may reveal unappreciated relationships between target epitope and clinical characteristics (30, 31). Because AnkG is an intracellular protein, anti-AnkG antibodies may not be directly pathogenic and may instead be reflective of autoreactive T-cell activity. However, both T- and B-cell infiltrates were detected in neuropathology.

Although intracellular, AnkG is a membrane-associated protein owing to its interaction with many AIS-enriched membrane proteins, an N-terminal palmitoyl moiety (32), and an adjacent solenoid ankyrin repeat domain that makes periodic contact with the plasma membrane (33). Using a synthetic blocking peptide, we were able to localize case 1's anti-AnkG epitope within amino acids 937–957, which lie in the linker region between the ankyrin repeat and the spectrin-binding domain (34). Replacement of AnkG's linker with the ankyrin B's linker region disrupts AnkG's association with the plasma membrane leading to an intracellular localization (35). As autoantibodies targeting other peripheral membrane proteins may be directly pathogenic (36, 37), it remains to be seen whether anti-AnkG^937−957^ antibodies directly alter AnkG function.

The AnkG^937−957^ blocking peptide also allowed us to identify a distinct population of subpial autoantibodies in the CSF of case 1. Although we were unable to identify the antigen, this subpial immune response may explain the different clinical phenotypes between case 1 and case 2. Alternatively, the difference may be because case 1 and case 2 target different AnkG target epitopes (30, 31) or anti-AnkG antibodies are rare epiphenomena of various neuroinflammatory processes.

Although AnkG autoantibodies have been reported in sera from healthy controls and individuals with Alzheimer's disease (38), these samples were not tested by TBIF, and the finding was not replicated (39). By TBIF, AIS-specific immunoreactivity has only been reported with CSF or serum from patients with TRIM46 (9, 29) or βIV-spectrin (12, 40) autoimmune neurological disorders. In contrast, we have not observed AIS immunostaining with CSF and serum from patients with systemic autoimmunity, cancer without neurological illness, or healthy controls (9).

Here, we report for the first time AnkG autoantibodies in the CSF of two patients with neurological illness. Because case 1 is living with HIV, we verified that AnkG autoantibodies were not secondary to his HIV status (8). Albeit extremely rare, these data suggest that, like myriad other antibodies targeting the AIS and NoR, AnkG autoantibodies are biomarkers of neurological autoimmunity.

## Limitations

Case 1 AnkG patient was inconsistently positive for NMDAR antibodies; therefore, we cannot rule out that this immune response contributed to his symptoms despite the atypical phenotype though that would be extremely unlikely clinically. It is possible that anti-AnkG occurred as a post-infectious autoimmune phenomenon in case 1, but the specific infection was not identified. Because CNS autoimmunity was not considered in case 2, she was not treated with immunotherapy. Moreover, both cases had different symptoms, suggesting that AnkG autoantibodies may not be biomarkers of a coherent neuroinflammatory syndrome. Furthermore, although we confirmed the presence of a distinct subpial antibody, we could not confirm its identity.

## Data availability statement

The datasets presented in this article are not readily available because of ethical and privacy restrictions. Requests to access the datasets should be directed to the corresponding authors.

## Ethics statement

The studies involving human participants were reviewed and approved by University of California San Francisco Institutional Review Board (IRB) numbers 13-12236 and 15-18425, and by Mayo Clinic Institutional Review Board (IRB) numbers 08-006647 and 08-007846. Written informed consent for participation was not required for this study in accordance with the national legislation and the institutional requirements. All procedures used in this study complied with federal guidelines and the institutional policies of the UCSF, Mayo Clinic, and Baylor College of Medicine Institutional Animal Care and Use Committees.

## Author contributions

CB: conceptual design, experimental design, tumor histology, IHC, CBAs, PhIP-Seq analysis, data interpretation, and writing. TN: CBAs, IHC, confocal microscopy, and writing. CC: neuropathological studies and editing. AH: clinical data and writing and editing. BA and IH: PhIP-Seq assays. RS: PhIP-Seq and writing and editing. KZ: clinical data collection and patient sample collection. TH and MR: AnkyrinG-deficient mice and writing and editing. AK and JD: PhIP-Seq database design. SS, JG, FC, and SeP: clinical data and editing. DD: tissue-based assay, clinical data, and writing and editing. MW and SaP: conceptual design, experimental design, data interpretation, and writing. All authors contributed to the article and approved the submitted version.

## Supplementary material

Supplementary Figure 1 Identification and validation of anti-AnkG antibodies in case 2 CSF by TBIF and CBA. **(A)** TBIF of murine cortex with CSF from case 2 shows AIS-like structures in the cortex (arrow), hippocampus, and the cerebellum (ml, molecular layer; gl, granule cell layer) and NoR-like structures in the midbrain. **(B)** 270 kDa rat AnkG-mCherry CBA. HEK 293T cells were transfected with AnkG-mCherry, fixed, permeabilized, and stained with CSF at a 1:100 dilution and DAPI. Scale bar = 10 μm.
