# Supplementary figures and images for: Dual ankyrinG and subpial autoantibodies in a man with well-controlled HIV infection with steroid-responsive meningoencephalitis: A case report
Source: Front Neurol. 2023 Jan 23;13:1102484. doi: 10.3389/fneur.2022.1102484 (PMC9900111; doi:10.3389/fneur.2022.1102484)

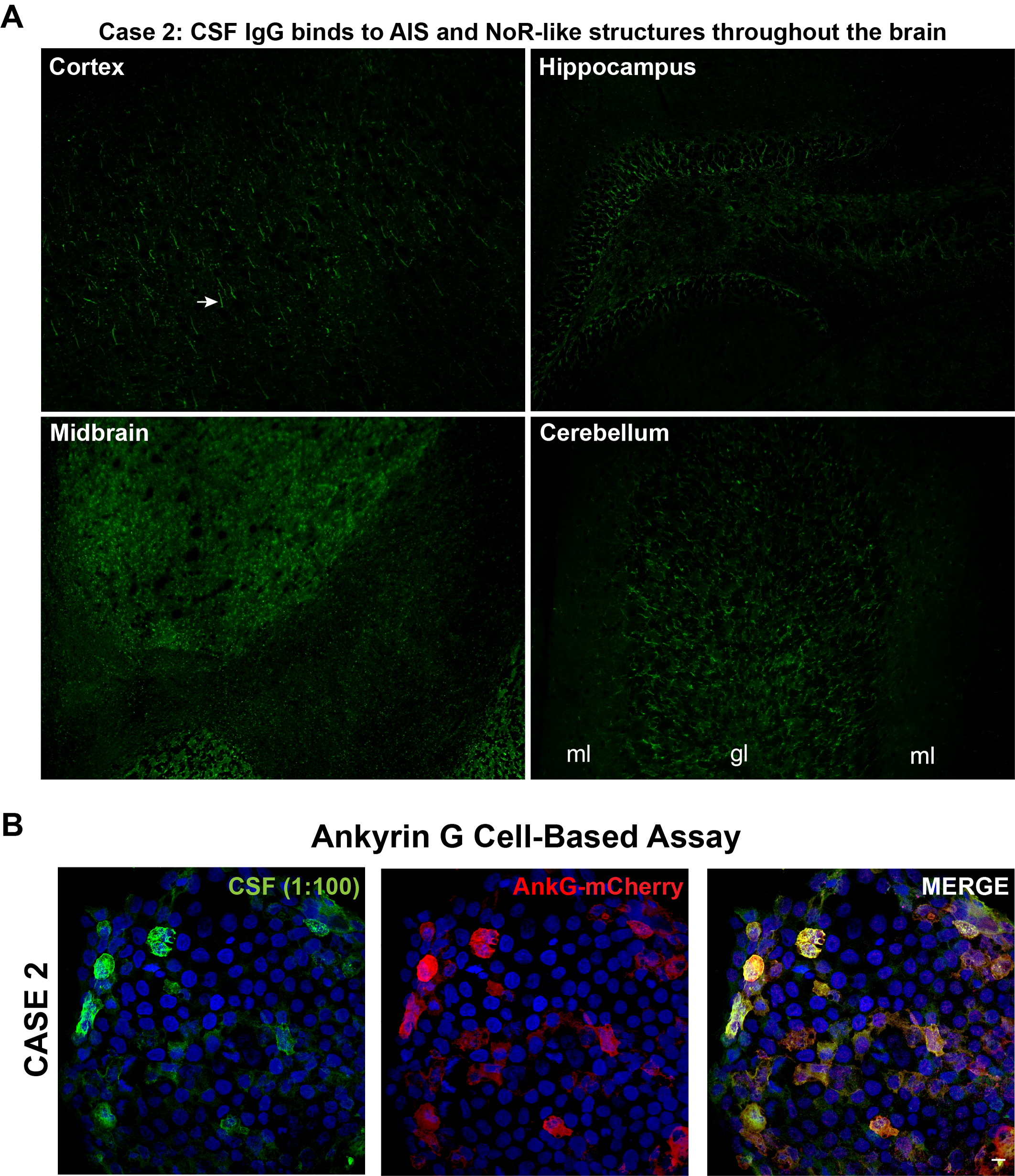

Supplement: Supplementary Figure 1 — Identification and validation of anti-AnkG antibodies in case 2 CSF by TBIF and CBA. (A) TBIF of murine cortex with CSF from case 2 shows AIS-like structures in the cortex (arrow), hippocampus, and the cerebellum (ml, molecular layer; gl, granule cell layer) and NoR-like structures in the midbrain. (B) 270 kDa rat AnkG-mCherry CBA. HEK 293T cells were transfected with AnkG-mCherry, fixed, permeabilized, and stained with CSF at a 1:100 dilution and DAPI. Scale bar = 10 μm. [file Image_1.PNG]
