# Supplementary material for: Dual ankyrinG and subpial autoantibodies in a man with well-controlled HIV infection with steroid-responsive meningoencephalitis: A case report
Source: Front Neurol. 2023 Jan 23;13:1102484. doi: 10.3389/fneur.2022.1102484 (PMC9900111; doi:10.3389/fneur.2022.1102484)
Supplement: Supplementary file 2 [file Data_Sheet_1.pdf]

Supplemental Materials and Methods

Patients

Written informed consent was obtained from healthy controls as part of an ongoing, open-ended study for autoantibody detection in individuals with unexplained neuroinflammation as approved by the Institutional Review Board of UCSF (IRB #13-12236). Written informed consent was obtained from case 1 for inclusion in a demonstration of precision medicine for the diagnosis of acute infectious diseases in hospitalized patients, which included the banking of clinical samples for future research as approved by the Institutional Review board of UCSF (IRB #15-18425).

Case 2 was identified in the course of a study of anti-TRIM46 antibody associated neurologic syndromes[9]. Of three samples that were negative for TRIM46, two were found to harbor antibodies to βIV spectrin[12]. The third case was negative for βIV and was included in the present study. This study was approved by Mayo Clinic Institutional Review Board (IRB) numbers 08-006647 and 08-007846.

Animals

Post-natal day 40 – 60 mice from the F1 cross of FVB (Jackson Laboratory, Cat. No. #001800) x C57BL/6J (The Jackson Laboratory, Cat. No. #000664) mice were used for initial tissue-based assays. All procedures used in this study complied with federal guidelines and the institutional policies of the University of California San Francisco Institutional Animal Care and Use Committee.

Post-natal day 14 (P14) *Avil-Cre:Ank3$^{fl/fl}$* nervous system tissues were generated by crossing *Avil-Cre* mice (#JAX:032536, RRID:IMSR_JAX032536) and *Ank3$^{fl/fl}$* mice (#JAX:029797, RRID:IMSR_JAX:029797) and housed at Baylor College of Medicine. All procedures with the *Avil-Cre:Ank3$^{fl/fl}$* were approved by the Baylor College of Medicine Institutional Animal Care and Use Committee.

Immunostaining

TBIF:

Mice were transcardially perfused with 4% paraformaldehyde (PFA) and brains post-fixed in 4% PFA for 4-6 hours. After 30% sucrose equilibration, brains were blocked in OCT and sectioned at 12 μm.

Sections were rehydrated in TBS-T (0.1% Tween-20), then blocked and permeabilized for one hour at room temperature using a blocking buffer containing 10% lamb serum and 0.1% Triton X-100, diluted in TBS-T. Next, sections were incubated in patient CSF at 1:4 to 1:100 or sera at 1:1000, with or without commercial antibodies, at 4°C overnight. The following day, sections were rinsed five times with TBS-T then incubated with fluorophore conjugated anti-IgG secondary antibodies (Jackson ImmunoResearch) diluted in blocking buffer for one hour at room temperature. Following five more TBS-T washes, sections were incubated with TrueBlack® Lipofuscin Autofluorescence Quencher (Biotium, #23007) diluted 1:20 in 70% ethanol for 10 minutes at room temperature. Sections were then washed several times using 1X

TBS, without detergent, before nuclear counterstaining using 1ug/mL DAPI. Lastly, stained sections were coverslipped using ProLong Gold Antifade (ThermoFisher, Cat No. P36930) or Prolong Diamond Antifade (ThermoFisher, Cat No. P36961).

Blocking Peptide against Putative AnkG Epitopes

NID-0908 CSF blocking peptide protocol has been published at https://www.protocols.io/edit/ankg-blocking-peptide-ucsf-csf-bgjvjun6.

Immunostaining of conditional knockout tissue

Tissues from *Avil-Cre:Ank3$^{fl/fl}$* mice were stained using the same procedure described above for tissue-based assays. Tissues were stained using CSF at 1:4, anti-Caspr 1:1000, anti-Ankyrin-G 1:500.

Phage Display Immunoprecipitation Sequencing (PhIP-Seq)

PhIP-Seq was performed as previously described.[10] As in a previous study[12], we used definitions from the European Molecular Biology Laboratory Bioinformatics Institute (EMBL-EBI) to define AIS (GO:0033268) and NoR (GO:0043194) proteins. Because AIS and NoR staining was observed in cases 1 and 2, only those proteins defined as both AIS and NoR proteins were included in the analysis (i.e. proteins defined as only either an AIS or a NoR protein were excluded).

For the protein-level analysis, the read counts per hundred thousand (rpK, = [absolute peptide read count x 100,000] / [total sample read count]) for all peptides mapping to a given AIS/NoR protein were summed. To calculate the fold change, the total read count was divided by the mean control rpK for that protein. For the peptide-level analysis, individual peptide rpKs were compared to the mean control rpK for that peptide and kept if fold change was ≥ 10. For the peptide-level heatmap, peptide enrichments were expressed as Z-scores to better represent the extremeness of the enrichment.

HEK293T Overexpression Cell-Based Assay (CBA)

Rat-AnkG-mCherry (https://www.addgene.org/42566/) and βIV-spectrin Σ1 and Σ6 cell-based assays were performed as previously described[12].

Synthetic Peptides

Control and blocking peptides were synthesized by Genscript at ≥ 98% purity.

Imaging

Panoramic images of mouse brain tissue immunofluorescence were acquired at 20X using a Zeiss Axio Scan Z.1 Slide Scanner. TBIFs and CBAs were imaged at 60X and 100X using a Nikon CSU-W1 spinning disk confocal microscope at the UCSF Nikon Imaging Center. Images were prepared using ImageJ (Version 2.1.0/1.53c).

Antibodies

**Primary Antibodies**			
**Name**	**Host Species**	**Vendor (Catalog No.)**	**Assay (Concentration)**
Anti-Ankyrin-G mAb, clone 106/36	mouse	NeuroMab (75-146)	ICC-IF (1:500)<br>IHC-F (1:500)
Anti-Caspr pAb	rabbit	Abcam (ab34151)	ICC-IF (1:1000)<br>IHC-F (1:1000)
Anti-Vimentin pAb	rabbit	Bioss (BS-0756R)	IHC-F (1:250)
**Secondary Antibodies**			
**Fluorophore**	**Specifications**	**Vendor (Catalog No.)**	**Assay (Concentration)**
Alexa Fluor 488	AffiniPure Donkey Anti-Human IgG (H+L)	Jackson ImmunoResearch (709-545-149)	ICC-IF (1:1000)<br>IHC-F (1:1000)
Alexa Fluor 594	AffiniPure Donkey Anti-Rabbit IgG (H+L)	Jackson ImmunoResearch (711-585-152)	ICC-IF (1:1000)<br>IHC-F (1:1000)

Cy™ 5	AffiniPure Donkey Anti-Mouse IgG (H+L)	Jackson ImmunoResearch (715-175-151)	ICC-IF (1:1000)<br>IHC-F (1:1000)
